# Mussel-Inspired Microgel Encapsulated NLRP3 Inhibitor as a Synergistic Strategy Against Dry Eye

**DOI:** 10.3389/fbioe.2022.913648

**Published:** 2022-06-01

**Authors:** Zhiwei Zha, Qiumeng Chen, Decheng Xiao, Chengjie Pan, Wei Xu, Liangliang Shen, Jianliang Shen, Wei Chen

**Affiliations:** ^1^ Key Laboratory of Ophthalmology, Optometry and Vision Science, School of Ophthalmology and Optometry, Eye Hospital, School of Biomedical Engineering, Wenzhou Medical University, Wenzhou, China; ^2^ Wenzhou Institute, University of Chinese Academy of Sciences, Wenzhou, China; ^3^ Oujiang Laboratory (Zhejiang Lab for Regenerative Medicine, Vision and Brain Health), Wenzhou, China

**Keywords:** ROS, polydopamine, dry eye disease, synergistic strategy, NLRP3

## Abstract

The inflammatory response mediated by oxidative stress is the main pathogenesis of dry eye, but clinical observations have shown that scavenging oxygen-free radicals alone has limited therapeutic effect. Moreover, the unique anatomy and physiology of the ocular surface result in low bioavailability of drugs, and higher concentration is required to achieve the desired efficacy, which, however, may bring systemic side effects. These problems pose a challenge, but the revelation of the ROS-NLRP3-IL-1β signaling axis opens up new possibilities. In this investigation, an NLRP3 inhibitor was successfully encapsulated in polydopamine-based microgels and used for dry eye treatment. It was demonstrated that the well-designed microgels exhibited good biocompatibility, prolonged drug retention time on the ocular surface, and effective inhibition of corneal epithelial damage and cell apoptosis. In addition, due to the synergistic effect, the NLRP3 inhibitor–loaded microgels could exert enhanced oxygen radical scavenging and inflammation-inhibiting effects at a lower dose than monotherapy. These findings suggest that polydopamine-based microgels have advantages as ocular surface drug delivery platforms and have promising applications in oxidative damage–related inflammatory diseases in synergy with anti-inflammatory drugs.

## Introduction

Dry eye disease (DED), a multifactorial chronic disorder of the ocular surface with a reported prevalence of 5–50%, affects millions of people worldwide, causing a marked impact on individual well-being (Mason et al., 2021). Especially at present, with the high-frequency use of video terminals, the incidence of DED is gradually increasing. DED is characterized by tear film instability and accompanied by hyperosmolarity, ocular surface inflammation, and damage, resulting in a variety of ocular discomforts and visual impairments (Bron et al., 2017). Tear hyperosmolarity can cause damage to the corneal and conjunctiva epithelial cells, leading to large production of inflammatory cytokines, which will further aggravate damage to the ocular surface epithelium and conjunctival goblet cells (Pflugfelder and de Paiva, 2017). These events will cause the release of damage-associated related pattern molecules, insufficient secretion of ocular surface mucins, and decreased tear film stability, with the interplay of these factors eventually leading to apoptosis (Pflugfelder and de Paiva, 2017; Reins et al., 2018; Baudouin et al., 2019). Current pharmacological treatments of DED include artificial tears, autologous serum, glucocorticoids, and non-hormonal immunosuppressants (Jones et al., 2017). Although artificial tears can temporarily relieve mild symptoms of DED patients and stabilize the tear film, the inflammation progression cannot be effectively controlled (Barabino et al., 2020). The application of autologous serum is limited due to the high requirement for preparation, susceptibility to contamination, and difficulty in long-term preservation (Mason et al., 2021). Long-term or improper use of glucocorticoids can cause serious complications such as elevated intraocular pressure and cataract (Roberti et al., 2020). While immunosuppressants can reduce the expression of inflammatory cytokines on the ocular surface, toxicity and side effects still limit their clinical application (Periman et al., 2020). Therefore, new strategies for effective and safe treatment of DED are still urgently required.

Regardless of the inducing mechanisms of DED, the immune inflammation of the ocular surface is regarded as the main cause. High osmotic pressure can trigger intracellular oxidative stress reaction, inducing intracellular outburst of reactive oxygen species (ROS) and irreversible damage to intracellular biological macromolecules (Wang et al., 2016; Favero et al., 2021). Moreover, it was demonstrated that oxidative damage of mitochondria can cause lacrimal gland dysfunction, leading to reduced tear production, increased tear ROS level, and eventually DED (Uchino et al., 2012). In addition, ROS can activate NOD-like receptor pyrin domain– containing protein 3 (NLRP3) in the corneal epithelium and further promote the secretion of interleukin-1β (IL-1β), leading to ocular surface inflammation (Zheng et al., 2014; Zheng et al., 2015). As one of the most prominent inflammasomes, NLRP3 regulates the generation of mature forms of IL-1β and is associated with inflammatory cell death (Zhang et al., 2021). Numerous studies have reported on the critical role of the NLRP3-IL-1β signaling axis mediated by ROS homeostatic imbalance during the inflammatory process with the development and progression of DED (Zheng et al., 2014; Lv et al., 2021; Wang B. et al., 2021). A comparison study indicated that compared with the healthy subjects, the level of ROS, NLRP3, and IL-1β in tears and conjunctival cells of DED patients was significantly improved, suggesting that ROS can further induce IL-1β release by activating NLRP3 expression (Zheng et al., 2015). These findings suggest that the ROS-NLRP3-IL-1β signaling axis may act as a potential therapeutic target for DED treatment. Currently, several NLRP3-related inhibitors have been identified (Jiang et al., 2017; Wong et al., 2018; Yu et al., 2018). Among them, MCC950, a sulfonylurea molecule, exhibits specificity in inhibiting the NLRP3 inflammasome, reducing IL-1β release and apoptosis without affecting other signaling pathways (Coll et al., 2019). Numerous studies have demonstrated that MCC950 has a good anti-inflammatory effect in various inflammatory diseases, such as cryopyrin-related syndrome, lung inflammation, renal fibrosis, hypertension, and myocardial infarction (Gao et al., 2019; Krishnan et al., 2019; Corcoran et al., 2021; Wang L. et al., 2021; Yoon et al., 2021). However, as far as we know, DED treatments using MCC950 have been seldom reported. We envision that the specific inhibition of NLRP3 by MCC950 combined with ROS scavenging will provide a synergistic strategy for DED treatment.

Nevertheless, topically administered ophthalmic solution drugs usually have poor bioavailability due to ocular surface barriers, such as limited corneal permeability, constant blinking, and rapid nasolacrimal drainage (Park et al., 2015). Accordingly, effective treatments require frequent application of drugs, leading to poor patient compliance and undesirable side effects. It has been well-recognized that drug carriers with high ocular surface adhesion and long retention time can effectively improve the bioavailability of ocular surface medication (Park et al., 2015; Lin et al., 2019). Mussel, a classic example of wet surface adhesion in nature, achieves excellent adhesion by secreting special adhesive proteins containing high levels of catechol amino acid (Yu et al., 2020). Several investigations have demonstrated that polydopamines, the mussel-inspired biomaterials with good biocompatibility and biodegradability (Li et al., 2020), exhibited excellent adhesion properties on almost any surface (Suneetha et al., 2019; Shao et al., 2020). For example, Jia et al. reported on the use of polydopamine microcapsules in prolonging the retention time of pesticides on leaves (Jia et al., 2014). In addition, the catechol moieties of polydopamines have been widely used to scavenge multiple types of ROS (Tang et al., 2019; Hu et al., 2020). It has been demonstrated that polydopamines can protect the brain from ROS-induced brain tissue damage during ischemic stroke injury (Liu et al., 2017), in addition to being applied in chronic inflammatory diseases (e.g., periodontitis) and acute inflammatory injuries (Bao et al., 2018; Zhao et al., 2018). However, to the best of our knowledge, combining the adhesiveness and ROS removal ability of polydopamines for ocular surface drug delivery is rarely reported.

**SCHEME 1:**
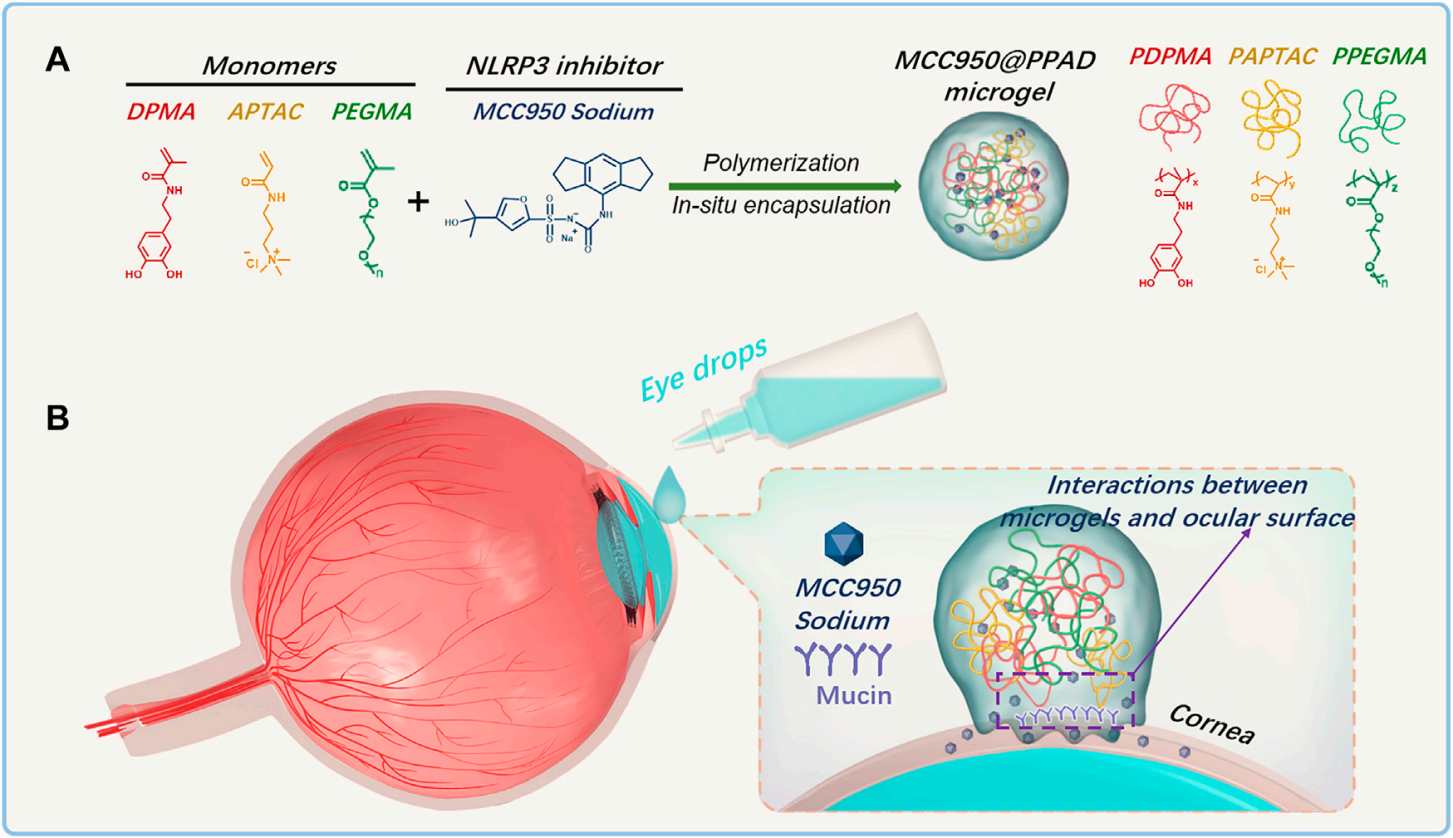
**(A)** Schematic diagram of the construction of the PPAD microgel and *in situ* encapsulation of MCC950 through radical copolymerization of PEGMA, APTAC, and DPMA; **(B)** Ocular surface retention time of MCC950@PPAD microgels can be enhanced due to the adhesiveness of polydopamines.

Considering the critical role of the ROS-NLRP3-IL1β signaling axis in the occurrence and development of DED, herein, a synergetic therapeutic strategy for DED treatment was proposed. As demonstrated in Scheme 1, a polydopamine-based microgel *in situ* loaded with MCC950 was constructed *via* radical copolymerization of poly (ethylene glycol) methacrylate (PEGMA), 3-acrylamidopropyl trimethylammonium chloride (APTAC), and N-(3,4-dihydroxyphenethyl) methacrylamide (DPMA), which was denoted as PPAD_x-y-z_ (x, y, and z referred to the molar ratio of PEGMA, APTAC, and DPMA, respectively). PEGMA was used to improve the biocompatibility of the microgel, while the positively charged APTAC was adopted for *in situ* encapsulation of the negatively charged MCC950 through electrostatic interaction. DPMA was expected to increase the ocular surface retention time and drug bioavailability due to its adhesiveness. Furthermore, DPMA will scavenge excessive ROS on the ocular surface and reduce the activation of the NLRP3 inflammasome, thus contributing to the synergistic treatment of DED combined with MCC950.

## Experimental

### Materials

MCC950 sodium and dihydroethidium (DHE) were purchased from MedChemExpress (MCE R; China). (3,4-dihydroxyphenethyl) methacrylamide (DPMA) was purchased from Macklin Biochemical Co., Ltd. (Shanghai, China). Poly (ethylene glycol) methacrylate (PEGMA) was purchased from Aladdin Reagent Co., Ltd. and passed through an Al_2_O_3_ column to remove the inhibitor before use. 3-acrylamidopropyl trimethylammonium chloride (APTAC, 75wt% in H_2_O) and lithium phenyl-2,4,6-trimethylbenzoylphosphinate (LAP, 98%) were obtained from Aladdin Reagent Co., Ltd and used without further purification. Fluorescein sodium (FS) was purchased from Abmole Co., Ltd. (United States). Scopolamine (Scop) was purchased from Sigma–Aldrich Co., Ltd. Human corneal epithelial cells (HCECs) were obtained from ATCC (Manassas, VA, United States). Dulbecco’s modified Eagle’s medium/nutrient mixture F-12 (DMEM/F-12; 1: 1 ratio), FBS, and insulin were purchased from Invitrogen, Gibco Co., Ltd. Triton X-100 was purchased from Sigma Co., Ltd. (Darmstadt, Germany). CM-H2DCFDA (General Oxidative Stress Indicator) was obtained from Invitrogen Molecular Probe Co., Ltd. Cell Counting Kit-8 (CCK-8) was purchased from Beyotime Biotechnology Co. (Shanghai, China). The *In situ* Cell Death Detection kit (TUNEL) was purchased from Roche Co., Ltd. (Germany).

### Characterization

Monomer conversions after polymerization were analyzed by ^1^H NMR spectroscopy on a QUANTUM-I-400MHz spectrometer using D_2_O as the solvent. The hydrodynamic diameter and polydispersity indexes of the microgels were analyzed using dynamic light scattering (DLS) measurements using a Malvern ZS90 with a He–Ne laser (633 nm, 4 mW) at a 90° angle. The morphology of the microgel was observed by using a transmission electron microscope (TEM) on a Jeol 200CX microscope (200 kV). To prepare the TEM samples, a small drop of the microgel solution (1 mg/ml) was carefully deposited onto a carbon-coated copper electron microscopy grid and vacuum-dried overnight. The *in vitro* release of FS from the microgels and the residual FS in rabbit tear samples were analyzed using fluorescence spectroscopy. Human corneal epithelial cells (HCECs) were observed on an inverted fluorescence microscope (Zeiss Axio Vert a3, Germany).

### Preparation and Characterization of MCC950@PPAD Microgels

PPAD microgels were prepared *via* photo-induced radical copolymerization of main monomers (PEGMA and APTAC) with the comonomer DPMA in an aqueous solution according to the procedures reported (Xue et al., 2017). Herein, the synthesis of MCC950@PPAD_3-7-1_ was described as a representative example. Briefly, PEGMA (0.257 g, 0.714 mmol), MCC950 sodium (2.84 mg, 6.65 μmol), LAP (14 mg, 47.7 μmol), and APTAC (0.345 g, 1.669 mmol) were added into a round-bottomed flask and dissolved in 10 ml of deionized water. After the mixed solution was bubbled with argon at 0°C for 30 min, the flask was sealed and exposed to illumination at 406 nm and stirred at 500 rpm. After 2 h of polymerization, 37 mg of DPMA (0.167 mmol) dissolved in 0.4 ml of ethanol was then injected into the polymerization mixture. After a few minutes, a milky suspension was observed (Supplementary Figure 1), indicating the formation of PPAD microgels. The polymerization continued for 4 h and the resultant microgels were purified by centrifugation. The detailed information of the polymerizations is summarized in Supplementary Table 1.

### *In Vitro* Drug Release From the PPAD Microgels

FS, a common fluorescent dye for cornea fluorescein staining, was selected as a model molecule to investigate the *in vitro* release behavior because of its similarity with MCC950, such as molecular weight, water solubility, and being negatively charged. Moreover, the concentration of FS can be conveniently detected by fluorescence spectroscopy. First, the purified FS@PPAD_3-7-1_ microgel was obtained and redispersed in a certain volume of ddH_2_O. Then, the FS@PPAD_3-7-1_ microgel dispersion was added into a dialysis tube with a molecular weight cut-off (MWCO) of 2000 and dialyzed against ddH_2_O or PBS with different ionic strengths (50 and 90 mM) at a stirring speed of 200 rpm. A certain volume of the dialyzate outside the dialysis tube was withdrawn at a certain time point and analyzed using fluorescent spectroscopy to monitor the *in vitro* release behavior.

### Drug Retention on the Ocular Surface

Herein, FS was also used as the model molecule to assess the ability of the PPAD microgel to improve drug retention on the ocular surface. Briefly, the rabbit eyes were washed with normal saline 1 hour before topical treatment with 50 μL FS solution (1%) or FS@PPAD microgels loaded with the equivalent amount of FS: 5, 15, 30, 45, 60, 90, 120, 150, and 180 min after topical treating, and tear samples were collected by immersing a square piece of filter paper with a size of 1 × 1 cm^2^ in the tear fluid for 10 s. Then, the filter paper was immersed in 500 μL of ddH_2_O, and FS was extracted from the filter paper using ultrasound. After proper dilution and filtration, the concentrations of FS in tear samples were analyzed by fluorescence spectroscopy.

### *In Vitro* Cytotoxicity and Ocular Histocompatibility Evaluation

The *in vitro* cytotoxicity of free MCC950, PPAD microgels, and MCC950@PPAD microgels on HCECs was evaluated by CCK-8 assay according to the manufacturer’s instructions and quantified on a microplate reader. Briefly, HCECs were suspended in DMEM-F12 medium supplemented with fetal bovine serum (10%, v/v) and insulin (5 μg/ml) and then seeded into a 96-well plate at a density of 5000 cells per well. After the HCECs were incubated at 37°C, 90% humidity, and 5% CO_2_ for 24 h to allow cell attachment, the original medium was replaced with a fresh medium containing different amounts of PPAD microgels, free MCC950 (0.05, 0.1, 0.2, 0.5, 1, 2, 5, and 10 μM), or MCC950@PPAD microgels loaded with an equal concentration of MCC950. After a further incubation of 24 h, the HCECs were gently washed two times with 100 μL prewarmed PBS, and the medium was replaced with 100 μL medium containing 10 μL CCK-8, followed by further incubation of 2 h. In addition, blank wells with no cells inside were also added with 100 μL of the medium containing 10 μL of CCK-8. The absorbance at 450 nm was recorded using an enzyme marker, and the viability of HCECs was determined based on the percentage of the absorbance of the treated cells compared to that of the untreated control HCECs.

For ocular histocompatibility evaluation, MCC950, PPAD_3-7-1,_ and MCC950@PPAD_3-7-1_ were instilled into the mice’s right eye five times a day for 7 days, and an equal volume of physiological saline solution was instilled into the left eye as the negative control. The mice were euthanized by cervical dislocation after 7 days and then the eyeballs were enucleated and fixed in 4% paraformaldehyde (PFA) overnight at 4°C. The samples were subsequently embedded in paraffin. Histological sections of tissues (cornea, conjunctiva, iris, and sclera) were stained with hematoxylin and eosin (HE) staining according to routine protocols and examined using a light microscope (Olympus, Japan).

### Evaluation of *In Vivo* and *In vitro* ROS Scavenging Capacity of PPAD_3-7-1_ and MCC950@PPAD_3-7-1_

To investigate the ability of PPAD_3-7-1_ or MCC950@PPAD_3-7-1_ to scavenge ROS, stained HCECs cells were treated with hyperosmotic stress by CM-H2DCFDA *in vitro* experiments. Mouse corneal tissues were treated by DHE *in vivo* experiments. For CM-H2DCFDA staining, HCECs were first seeded in a black 96-well plate at a density of 5 × 10^3^ cells per well. After the cells were incubated for 24 h, the medium was replaced with DMEM-F12 without FBS containing 90 mM NaCl to create a highly permeable environment (500 mOsm) (Zheng et al., 2018). As the control group, HCECs were also cultured under physiological isotonic conditions. Then, different concentrations of PPAD_3-7-1_ microgels were added into the wells so that the final concentrations of the PPAD_3-7-1_ microgels were 0.01 mg/ml, 0.1 mg/ml, and 1 mg/ml. After the cells were cultured for 18 h under the hyperosmotic environment, intracellular ROS levels were measured using a reactive oxygen species detection kit (Roche) according to the manufacturer’s instructions. Briefly, the cells were gently washed two times using 100 μL of prewarmed PBS, added with 100 uL of CM-H2DCFDA reaction solution diluted by PBS at a concentration of 10 μM and then incubated in an incubator saturated with 5% CO_2_ and 90% humidity. After 45 min, the CM-H2DCFDA reaction solution was replaced with PBS, and the HCECs were observed under an inverted phase contrast fluorescence microscope (ZEISS Axio Observer3) after two-time gentle washing with 100 μL PBS. For DHE staining, DED mice were treated with MCC950, PPAD_3-7-1_, MCC950/PPAD_3-7-1_ (simple mixture of MCC950 and PPAD_3-7-1_), and MCC950@PPAD_3-7-1_ three times a day for 5 days. The mice were euthanized by cervical dislocation after 5 days and then the eyeballs of the mice were enucleated and embedded with OCT. Subsequently, the embedded samples were cut into 10-μM sections and washed with cold PBS. Then, the cornea sections were incubated with 10 μM fluorescent dye DHE at 37°C for 40 min in a humidified chamber and protected from light. The sections were observed under 20x magnification using a laser confocal microscope (LSM 880 NLO with AiryScan, Zeiss, Germany), and the mean fluorescence intensity was analyzed by using ImageJ software (ImageJ 1.8; NIH, Bethesda, MD, United States).

### Experimental dry eye Disease Animal Model Preparation

C57BL/6j female mice (6–8 weeks, 18–22 g) were obtained from Jiesijie Experimental Animal Co. (Shanghai, China) and maintained in the experimental animal center of Wenzhou Medical University under the regulation of the Code of Conduct for Care and Use of Laboratory Animals. Animal experiments were approved by the Ethical Review Committee for Experimental Animals of Wenzhou Medical University. Before the experiments, the mice were kept in the animal center for 1 week for acclimation and medically examined to exclude unhealthy ones, especially with ocular pathological changes. The immune DED animal model was induced by subcutaneous injection of 200 μL Scop (2.5 mg/ml) three times per day for 5 days (Chen et al., 2013a). Subcutaneous injections were administered at 9:00 a.m., 13:00 p.m., and 19:00 p.m. Afterward, the mice were topically treated with 10 µL MCC950 (10 μM), 10 µL MCC950@PPAD microgel (with 10 μM MCC950), or 10 µL PPAD microgel three times per day for 5 days. As for the animal experiments shown in *Supplementary Material*, dry eye mice were treated with free MCC950 solution (1 μM, 10 μM, 100 μM) four times a day for 5 days. All dry eye mice were kept at 25°C with 13.1 ± 3.5% humidity and 2.2 ± 0.2 m/s airflow.

### Corneal Fluorescein Sodium Staining Score

After topical treatment for 5 days, the degree of corneal damage was evaluated using corneal fluorescein staining (CFS). CFS was observed using slit-lamp microscopy 2 min after 2 μL FS solution (2 mg/ml) was instilled into the conjunctiva sac. The scores were calculated according to the National Eye Institute (NEI) scale.^42^ Briefly, an experienced ophthalmologist graded them based on the number of positive fluorescein staining points in five areas of the cornea and calculated the total score. Each area was scored according to the following: grade 0 = no significant corneal staining; grade 1 = less than five fluorescent dots; grade 2 = 6–15 fluorescent dots; grade 3 = 16–30 fluorescent dots; and grade 4 = more than 30 fluorescent dots (Lemp, 1995).

### Methods for TUNEL Assay

After the mice were euthanized by cervical dislocation, the eye balls were carefully isolated, soaked in Optimum Cutting Temperature (OCT) media, and then frozen in liquid nitrogen. Ten-μm sagittal sections of eye tissues were prepared using a cryostat (HM 500; Micron, Waldorf, Germany) and stored at −80°C before use. Cell apoptosis of these tissue sections was detected by TUNEL (terminal deoxynucleotidyl transferase–mediated dUTP nick-end labeling) staining using an *In Situ* Cell Death Detection Kit (Roche, Mannhein, Germany) according to the manufacturer’s instructions. After 20 min of incubation at room temperature, the sections were washed three times with PBS and fixed with 4% paraformaldehyde for 20 min. Then, the sections were transferred into a thermostat at 4°C and permeabilized with 0.5% Triton X-100 for 5 min. Next, the sections were washed three times with PBS to remove Triton X-100. TUNEL staining was performed by adding 50 μL of the staining agent onto the sections, followed by an incubation of 1 h at 37°C. In addition, the sections were counterstained with DAPI for 1 min. The specimens were embedded in a mounting medium (Invitrogen, Oregon, United States), and their fluorescence images were recorded on a Zeiss confocal microscope with a krypton/argon and He–Ne laser (Carl Zeiss Meditec, Sartrouville, Germany) under an excitation wavelength of 405 nm for DAPI and 488 nm for TUNEL. The apoptotic cells were observed under 20x magnification using a laser confocal microscope (LSM 880 NLO with AiryScan, Zeiss, Germany).

### *In Vivo* Expression of Inflammation-Related Factors

The expression of interleukin-6 (IL6), NLRP3, and IL-1β in the ocular surface tissues of C57BL/6j mice at protein level was examined by Western blot. After treatment of DED mice for 5 days, the mice were euthanized and the ocular surface tissue was subsequently cut into small pieces and immediately lysed with 100 μL of PMSF lysis buffer (100 mM), followed by ball milling for 8 min. The proteins in the supernatant were collected following centrifugation at 16,000 g for 10 min at 4°C. Protein concentrations were measured by a BCA assay kit (Pierce). The proteins (20–30 μg) were mixed with 5X SDS sample buffer and loaded onto SDS-PAGE. After electrophoresis, the separated proteins were transferred to PVDF membranes. The membranes were blocked with 5% milk powder in TBST for 2 h at room temperature. After blocking, the membranes were incubated overnight at 4°C with rabbit anti-IL-1β (1:1000, Abcam, #ab234437), mouse-anti-NLRP3 (1:1000, adpiogene, #AG-20B-0014-C100), rabbit anti-IL6 (1:1000, cell signaling technology, #12912S), and rabbit α/β-tubulin Antibody (1:3000, Cell signaling technology, #2148). Afterward, the membranes were rinsed with TBST and then incubated with HRP-labeled rabbit IgG (1:3000, Biosharp, #BL003A) OR mouse IgG (1:3000, Biosharp, #BL001A) for 2 h. Protein bands were visualized using an enhanced chemiluminescence (ECL) kit and imaged by an AI680 ultra-sensitive multifunctional analyzer. Protein expression was quantified based on the ratio of the target protein and tubulin.

The mRNA expression level of IL-1β, NLRP3, and IL6 in the ocular surface tissue of DED mice was evaluated using the reverse-transcription polymerase chain reaction (RT-PCR). The detailed information on these experiments can be found in our previously published articles.^28^ The specific primers for mouse IL-1β, NLRP3, and IL6 genes were designed using the NCBI Primer-Blast (NCBI Web site) and are listed in Supplementary Table 2, which had 55–63% GC content. GAPDH was used as the internal reference gene. The expression levels of the target genes were normalized with the expression levels of the internal reference genes according to the manufacturer’s instructions.

### Statistical Analysis

Each experiment was performed three times, and all data were recorded as means ± standard deviation. Significant differences among groups were determined by one-way analysis of variance (ANOVA) with the level of significance set at *p* < 0.05.

## Results and Discussion

### Preparation and Characterization of MCC950@PPAD Microgels

**FIGURE 1:**
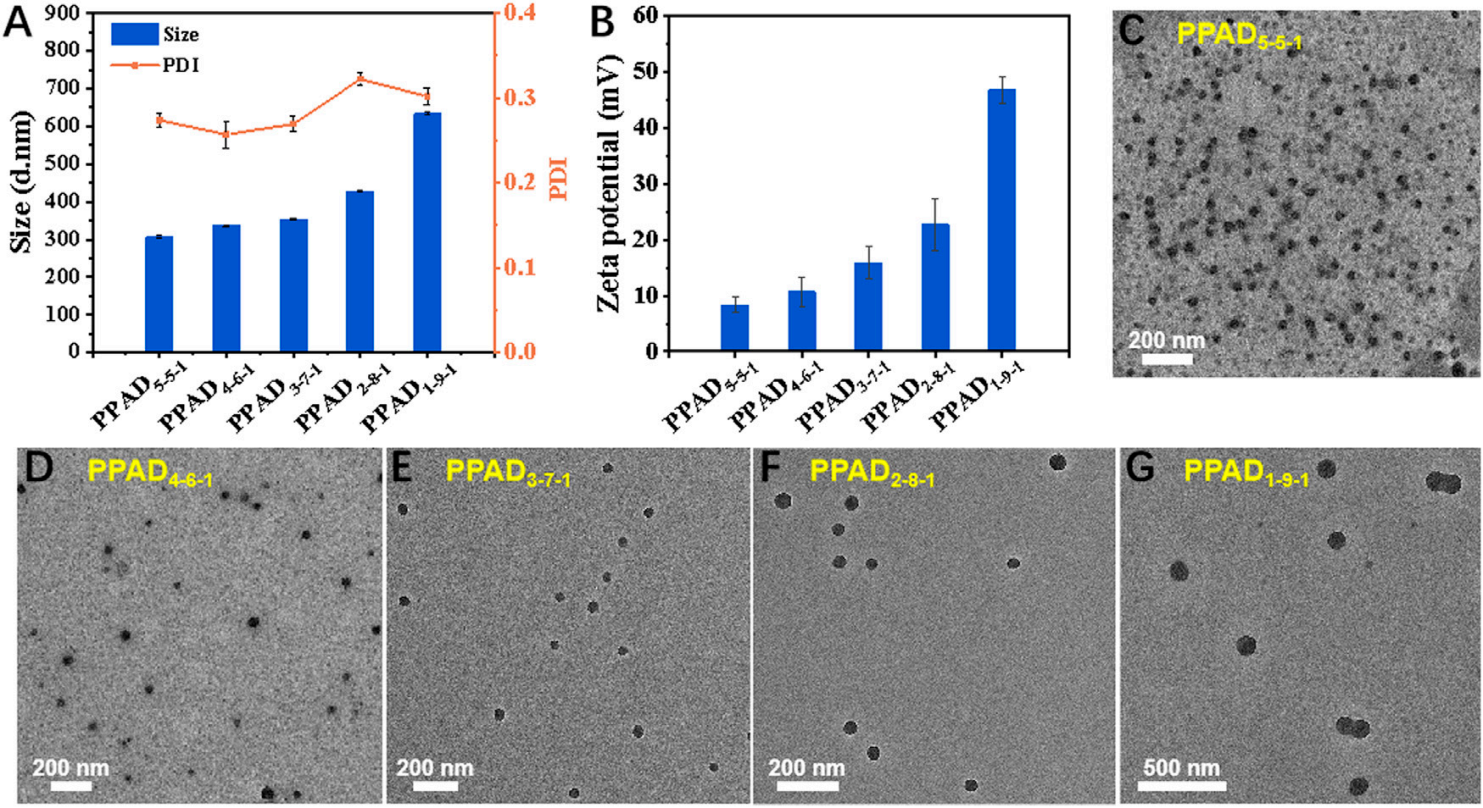
**(A)** Average value of apparent hydrodynamic diameter (*D*_h_, app), polydispersity index (PDI), **(B)** zeta potential, and morphology of the PPAD microgels, **(C–G)** TEM micrographs of the PPAD microgels.

As demonstrated in Scheme 1, three kinds of monomers (PEGMA, APTAC, and DPMA) were adopted to prepare PPAD microgels. As well-demonstrated in a previous report (Xue et al., 2017), the comonomer DPMA was needed as a cross-linker for the preparation of microgels. However, increasing the feed ratio of DPMA to the main monomers would cause low colloidal stability and coagulation in aqueous solution. When the feed ratio of DPMA to the main monomers was from 1/20 to 1/8, stable microgels were obtained. Thus, in our study, the molar ratio of DPMA to the main monomers (PEGMA and APTAC) was set to be 1:10. Since the cationic monomer APTAC was responsible for *in situ* encapsulation of MCC950, the content of APTAC is a critical factor. Although the high content of APTAC can contribute to the high encapsulation capacity of MCC950, too many positively charged polymers may lead to cytotoxicity and ocular surface damage. Moreover, the electrostatic repulsion among cationic PAPTAC polymers also influences the size of the microgels. Thus, the impact of APTAC content on the size and zeta potential of the PPAD microgels was first investigated. The feed molar ratio for the synthesis of the PPAD microgels is summarized in Supplementary Table 1.^1^H NMR spectra in Supplementary Figure 2 revealed near-quantitative monomer conversions. The DLS results in Figure 1A indicated that the average hydrodynamic diameter of the PPAD microgels gradually increased with the increase of the APTAC content. This is probably because the increased content of APTAC improved the electrostatic repulsion among PAPTAC polymers, leading to the swelling of the microgels. It is also reasonable that the zeta potential of the microgels gradually increased with the increase of the APTAC content. The morphology of the microgels was observed by TEM and is shown in Figures 1C–G. Although the TEM micrographs demonstrated that microgel size increased with the increase of APTAC content, the average diameter revealed by TEM was significantly lower than that determined by DLS, which is also probably due to electrostatic repulsion among PAPTAC polymers. Since the microgels were dispersed in an aqueous solution during DLS characterization, the sufficiently ionized PAPTAC polymer chains resulted in strong electrostatic repulsion and a high degree of swelling. On the contrary, because the microgel samples were dehydrated during TEM analysis, the PAPTAC polymers were not ionized and the strong electrostatic repulsion disappeared, leading to significant shrinkage and size decrease. The drug encapsulation efficiency in PPAD microgels was evaluated by using FS as the model molecule in place of MCC950. After centrifugation of the FS@PPAD microgels, the concentration of FS in the supernatant was analyzed by fluorescence spectroscopy to confirm the encapsulation efficiency. As summarized in Supplementary Table 1, high encapsulation efficiency (more than 80%) was achieved for all the PPAD microgel samples. Combining the size, zeta potential, morphology, and drug encapsulation efficiency, the PPAD_3-7-1_ microgel with a mean *D*_h_ of 356 nm and zeta potential of + 15.9 mV was selected as the optimal sample for the following *in vitro* and *in vivo* experiments.

### *In Vitro* Drug Release From the PPAD_3-7-1_ Microgels

**FIGURE 2:**
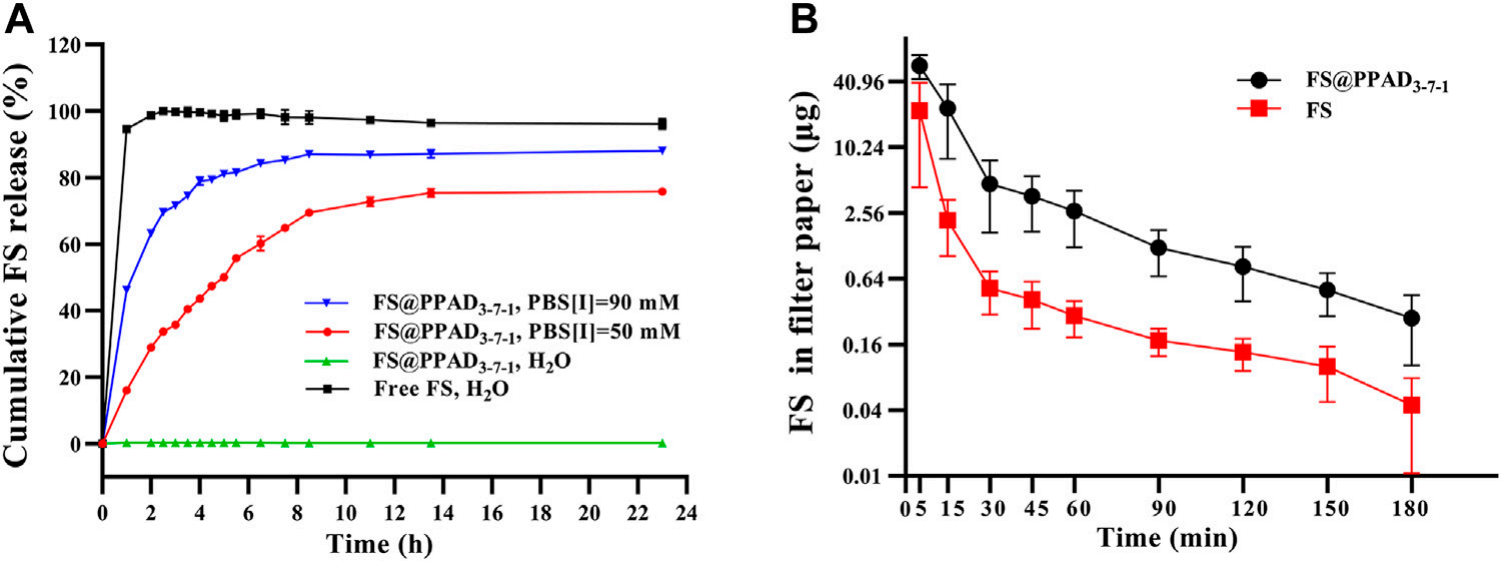
**(A)** *In vitro* cumulative release of FS from PPAD_3-7-1_ microgels under different conditions. **(B)** FS in a filter paper after a single instillation of free FS or FS@PPAD_3-7-1_ microgels.

Herein, FS was used as the model molecule to evaluate the release behavior of MCC950 from the PPAD_3-7-1_ microgel under different conditions. The release of free FS in ddH_2_O as a control was also carried out. As shown in Figure 2A (the black line), the cumulative release of free FS in ddH_2_O rapidly reached more than 90% in the first hour. In contrast, nearly negligible FS release from the PPAD_3-7-1_ microgel in ddH_2_O was observed because FS was stably encapsulated in PPAD_3-7-1_ microgels through electrostatic interaction. Thus, it can be reasonably anticipated that the FS release from the PPAD_3-7-1_ microgel can be accelerated by ionic strength. As indicated in Figure 2A (the red line), 19.34% of cumulative FS release from the PPAD_3-7-1_ microgel under the ionic strength of 50 mM was achieved within 1 h and reached 72.7% after 11 h. Furthermore, FS release from the PPAD_3-7-1_ microgel under the ionic strength of 90 mM was significantly improved (the blue line). It has been reported that tear hyperosmolarity is one of the core pathogenesis mechanisms of DED. Moreover, a culture medium containing NaCl (90 mM) was usually utilized to create a highly permeable condition. Thus, based on the abovementioned release results, we envision that efficient drug release can be achieved under hyperosmotic conditions of the ocular surface in the dry eye model.

### Ability of PPAD_3-7-1_ Microgels to Prolong the Ocular Surface Retention Time of Drugs

Due to sealed anatomical features of the eyes and ocular surface barriers, more than 90% of topically-applied drugs can be rapidly washed away within minutes, leading to poor bioavailability (Davis et al., 2004; Li et al., 2007). Thus, in this work, the polydopamine-based microgels are expected to improve the drug bioavailability by improving ocular surface adhesion and retention. Accordingly, undesired side effects of MCC950 can be avoided by reducing the dosage (Wang F. et al., 2021). Herein, the ocular surface retention was evaluated by detecting the concentration of FS in tear samples after topical treatment. The tear samples were collected using a filter paper at different time points after topical treatments. As shown in Figure 2B, 30 min after the rabbits were topically treated with 50 μL of PPAD_3-7-1_ microgels containing 10 mg/ml FS, a significantly (*p* < 0.05) higher amount of FS was detected in tear samples than in the rabbits treated with 50 μL of free FS (10 mg/ml), proving the ability of PPAD_3-7-1_ microgels to prolong ocular surface retention time.

### Cytotoxicity of MCC950@PPAD_3-7-1_ Microgels and Ocular Histocompatibility Evaluation

**FIGURE 3:**
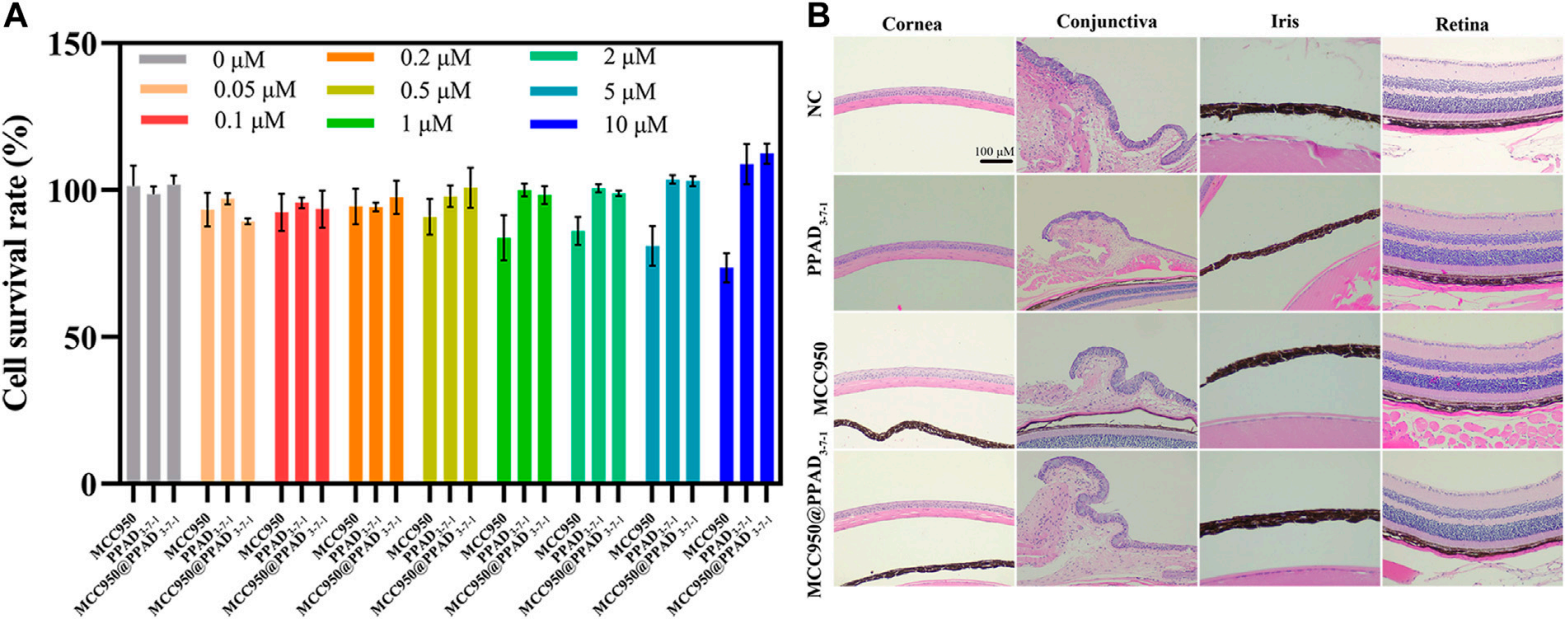
**(A)** Cytotoxicity of free MCC950, PPAD_3-7-1_ microgel, and MCC950@PPAD_3-7-1_ microgel. The concentrations of free MCC950 and PPAD_3-7-1_ microgel were equal to those of MCC950@PPAD_3-7-1_ microgels. The concentration of MCC950 varied from 0 to 10 μM. **(B)** Histopathology microscopy of ocular tissues, including cornea, conjunctiva, iris, and retina, after instilment with 0.9% (w/v) NaCl solution (NC), MCC950, PPAD_3-7-1_, and MCC950@PPAD_3-7-1_ five times a day for 7 days.

As MCC950@PPAD_3-7-1_ is a novel drug for the treatment of DED, it was necessary to evaluate the ocular histocompatibility of MCC950, PPAD_3-7-1_, and MCC950@PPAD_3-7-1_. The cytotoxicity of MCC950, PPAD_3-7-1_ microgels, and MCC950@PPAD_3-7-1_ microgels was evaluated using the CCK-8 assay. As shown in Figure 3A, free MCC950 at concentrations less than 0.5 μM was well-tolerated (more than 80% of HCECs survived after 24 h of incubation), while significant cell growth inhibition was observed with the MCC950 concentration up to 10 μM. On the contrary, when treated with the MCC950@PPAD_3-7-1_ microgel loaded with 10 μM of MCC950, HCEC survival was approximately 103%, suggesting that loading MCC950 into PPAD_3-7-1_ microgels significantly reduced the cytotoxicity of MCC950. Furthermore, when treated with PPAD_3-7-1_ microgels, high HCEC survival was also achieved, probably because the abundant dopamine moieties of the PPAD_3-7-1_ microgels scavenged excessive ROS, thus providing effective protection of HCECs. As shown in Figure 3B, HE stained slides were observed under a light microscope to evaluate the tissue’s (cornea, conjunctiva, iris, and retina) structure and integrity for histocompatibility detection. The eyes showed no significant pathologic changes when treated with all formulations. As indicated in Figure 3A, 10 μM MCC950 inhibited cell proliferation in the CCK-8 assay. However, when it was applied to the ocular surface, MCC950 could be rapidly cleared from the ocular surface through the lacrimal drainage system and diluted by tear fluid due to its good water-solubility. Therefore, when 10 μM MCC950 was dropped into the ocular surface, the actual concentration of MCC950 was much lower than 10 μM. Moreover, because of the intact morphological structure of the cornea, conjunctiva, iris, and retina, no inflammatory cell infiltration was observed after treatment with PPAD_3-7-1_ and MCC950@PPAD_3-7-1_. This indicates that PPAD_3-7-1_ and MCC950@PPAD_3-7-1_ exhibit excellent ocular biocompatibility.

### Ability of PPAD_3-7-1_ Microgels to Scavenge ROS Produced in Human Corneal Epithelial Cells Under Hyperosmotic Conditions

**FIGURE 4:**
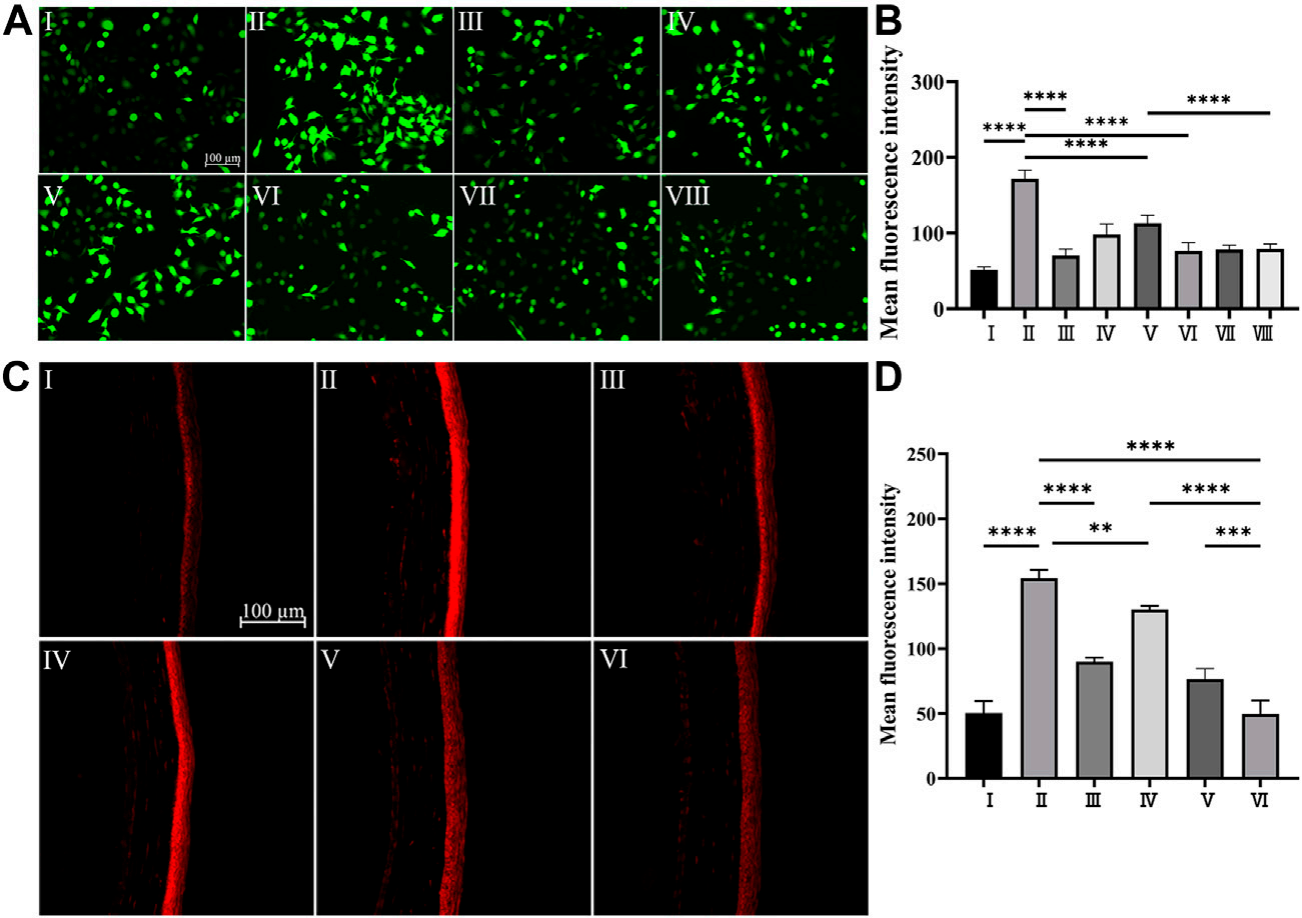
**(A,B)** *In vitro* ROS scavenging capacity of PPAD_3-7-1_ and MCC950@PPAD_3-7-1_, Groups I–II are designated as NC and HS, respectively. Groups III–V are designated as HS + PPAD_3-7-1_ microgels (the concentrations of PPAD_3-7-1_ microgels are 1 mg/ml, 0.1 mg/ml, and 0.01 mg/ml, respectively). Groups III–V are designated as HS + MCC950@PPAD_3-7-1_ (the concentrations of PPAD_3-7-1_ correspond to III-VIII). **(C,D)** *In vivo* ROS scavenging capacity of MCC950@PPAD_3-7-1_. Groups I–VI are designated as NC, Scop, Scop + PPAD_3-7-1_, Scop + MCC950, Scop + MCC950/PPAD_3-7-1_, and (iv) Scop + MCC950@PPAD_3-7-1_. Data are shown as mean ± SD, *n* = 4, **p < 0.01, and ****p < 0.0001.

Under physiological conditions, intracellular ROS levels remain at equilibrium due to the redox system which regulates the production and the clearance of ROS through enzymatic or nonenzymatic reactions (Sies, 2015). As one of the main features of DED, tear film hyperosmolarity can cause excessive production of ROS (Chen et al., 2013b), which further triggers the inflammatory cascade effect of DED development (Seen and Tong, 2018). Plenty of research has demonstrated that polydopamine-based biomaterials exhibited a good scavenging effect on a wide range of ROS due to their catechol moiety (Hu et al., 2020). As shown in Figure 4A Ⅱ, when HCECs were exposed to hyperosmotic stress, overproduction of ROS was observed compared with that of HCECs under physiological conditions (Figure 4A Ⅰ). However, as shown in Figure 4A Ⅲ-Ⅴ, the overexpressed ROS in HCECs induced by hyperosmotic stress can be effectively reduced by PPAD_3-7-1_ microgels. The quantitative analysis of the fluorescence intensity shown in Figure 4B was well-consistent with the fluorescent images. Moreover, the intracellular ROS can be restored to physiological level when treated with 1 mg/ml PPAD_3-7-1_ microgels (Figure 4A Ⅲ), suggesting that PPAD_3-7-1_ microgels can act as an excellent ROS scavenger. Interestingly, when MCC950 was loaded in PPAD_3-7-1_ microgels, the ability to inhibit ROS production was further enhanced (Figure 4A Ⅵ-Ⅷ). It is known that inflammatory response and increased ROS production are a mutually reinforcing process; oxidative stress can lead to increased production of inflammatory factors, while inflammation can also promote ROS production. Polydopamine scavenges overproduced ROS by chemical reactions; thus, the inhibitory effect of PPAD_3-7-1_ on ROS shows dose-dependence (Figure 4B), while MCC950, an NLRP3 inhibitor, reduces ROS production by inhibiting the inflammatory response (Supplementary Figure 4). When PPAD_3-7-1_ is loaded with MCC950, inflammation that is not completely inhibited by PPAD_3-7-1_ is further suppressed by MCC950, resulting in better ROS scavenging. To further verify the effect of MCC950@PPAD_3-7-1_ on ROS production in the corneal tissue of DED mice, DHE staining is used. The DHE staining of mouse corneal tissues showed similar results in Figures 4C,D, with MCC950@PPAD_3-7-1_ having the best ROS scavenging effect (Figure 4C Ⅵ). Collectively, these results suggest that MCC950 can inhibit ROS production in an anti-inflammatory manner, while PPAD_3-7-1_ microgels directly consume ROS through its polyphenol structure. Moreover, when MCC950 is loaded in PPAD_3-7-1_ microgels, a better ROS scavenging effect can be achieved due to the synergistic therapeutic effect.

### *In Vivo* Curative Effect Against DED

**FIGURE 5:**
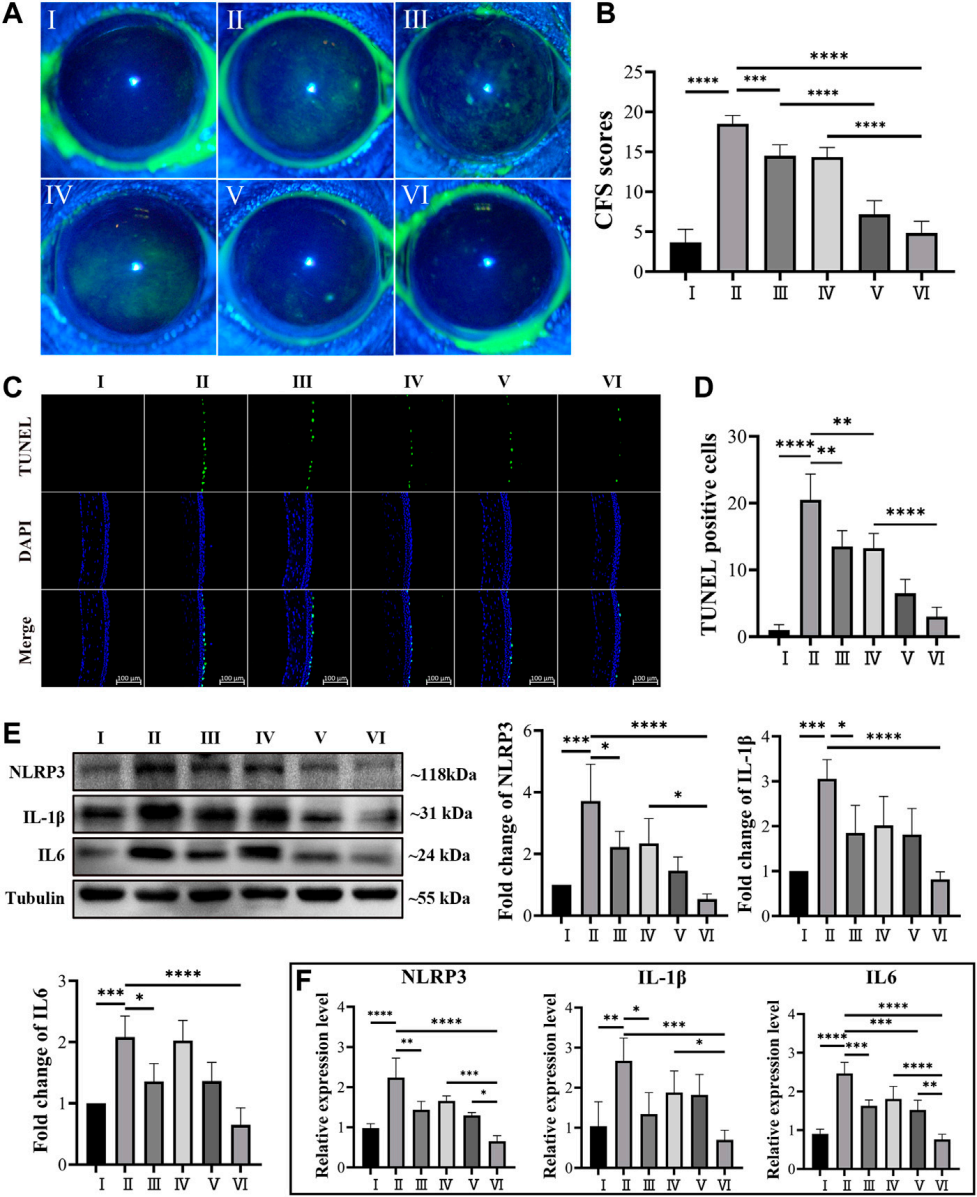
**(A,B)** Corneal fluorescein sodium staining (CFS) scores in in DED mice. **(C,D)** Representative images of TUNEL staining and number of TUNEL-positive cells in the DED mice corneal epithelium. **(E)** Western blot analysis revealed the protein level of IL-1β, NLRP3, and IL6 using tubulin as the internal control, and **(F)** mRNA expression level of IL-1β, NLRP3, and IL-6 in the ocular surface tissue of DED mice. (Groups I–VI are designated as NC, Scop, Scop + PPAD_3-7-1_, Scop + MCC950, Scop + MCC950/PPAD_3-7-1_, and Scop + MCC950@PPAD_3-7-1_, respectively.) Data are given as the mean ± SD (*n* = 4), *p < 0.05, **p < 0.01, ***p < 0.001, and ****p < 0.0001.

Five days after topical treatment of different MCC950 formulas, the corneal epithelial damage was assessed by using corneal fluorescein staining and imaged using a slit lamp under cobalt blue light (Schmidl et al., 2020), which is one of the most important bases to evaluate the severity of DED (Osei et al., 2020; Wolffsohn et al., 2017). The corneal epithelial damage or defect was statistically scored based on the guidelines of the National Eye Institute/Industry Workshop, United States . As shown in Figure 5A Ⅰ, the corneal epithelium of healthy mice exhibited a clean and blue profile with negligible green dots. After 5 days of Scop treatment, dense and scattered green dots were observed, indicating severe corneal epithelial damage (Figure 5A Ⅱ). Figure 5A Ⅳ demonstrated that no obvious curative effect was observed when topically treated with free MCC950 aqueous solution (10 μM), probably due to the short retention time on the ocular surface (Lemp, 1995). On the contrary, topical application of the MCC950@PPAD_3-7-1_ microgel significantly decreased the green dots of the cornea, and no significant difference was observed compared with the healthy cornea (Figure 5A Ⅵ). The curing effect of PPAD_3-7-1_ microgels alone shown in Figure 5A Ⅲ proved that the ROS scavenging ability of PPAD_3-7-1_ also contributed to the curing effect, confirming the synergistic treatment effect of MCC950@PPAD_3-7-1_ microgels. In addition, the curing effect of a simple mixture of PPAD_3-7-1_ and MCC950 was also evaluated, and a significantly lower curative effect was found (Figure 5A Ⅴ), demonstrating that a superior curative effect can be achieved when MCC950 was *in situ* encapsulated in PPAD_3-7-1_ microgels. When MCC950 was simply mixed with PPAD_3-7-1_ microgels, part of the MCC950 was not encapsulated inside the microgels and could be rapidly washed away after topical treatment, leading to a compromise of the curative effect.

The apoptosis of the corneal epithelium, an important part of DED pathogenesis (Kessal et al., 2021), was further investigated by using TUNEL assay. Frozen sections of corneal tissues were processed using an apoptosis detection kit and photographed by laser confocal microscopy. As shown in Figures 5C,D, only a small number of TUNEL-positive cells were found in the corneas of the NC mice, while a large number of TUNEL-positive cells were observed in the corneas of the mice subcutaneously injected with Scop for 5 days. This is consistent with that observed in previous studies (Park et al., 2018). When topically treated with PPAD_3-7-1_ microgels, free MCC950, or a simple mixture of PPAD_3-7-1_ microgels and MCC950, the number of TUNEL-positive HCECs was slightly decreased, indicating that the ocular surface damage was slightly alleviated. On the contrary, the apoptosis of the corneal epithelium was significantly inhibited after the topical treatment of MCC950@PPAD_3-7-1_ microgels, indicating that a superior curative effect can be achieved when MCC950 was *in situ* encapsulated in PPAD_3-7-1_ microgels rather than simple mixing. As shown in Supplementary 3, 5, effective inhibition of corneal epithelium apoptosis was achieved when treated with free MCC950 at a concentration up to 100 μM, which, however, may cause undesirable side effects due to the high dose. The superior therapeutic effect of MCC950@PPAD_3-7-1_ microgels can be attributed to the synergistic effect by combining the ROS scavenging effect of PPAD_3-7-1_ microgels and specific inhibition of NLRP3 by MCC950. In addition, the excellent adhesiveness of the PPAD_3-7-1_ microgels significantly improved the bioavailability of MCC950 by prolonging the ocular surface retention time.

### Effect of MCC950@PPAD_3-7-1_ Microgels on Inflammatory Cytokine Expression in Ocular Surface

To further elucidate the therapeutic effects of MCC950@PPAD_3-7-1_ microgels, real-time fluorescence quantitative PCR and Western blot experiments were used to evaluate the expression of several inflammation-related cytokines of DED at mRNA and protein levels (Ma et al., 2021; Zhang et al., 2021). As shown in Figure 5F, the mRNA expression level of several proinflammatory cytokines, such as IL6, NLRP3, and IL-1β, had significantly increased after Scop treatment (Kessal et al., 2021), suggesting the successful induction of ocular surface inflammation. This is consistent with the corneal fluorescein staining and TUNEL assay. The inhibition of expression at the mRNA level by free MCC950 exhibited a dose-dependent manner, which is shown in Supplementary Figure 6. Although the PPAD_3-7-1_ microgel alone or free MCC950 exhibited an inhibitory effect on mRNA expression of these inflammatory factors, topical treatment of MCC950@PPAD_3-7-1_ microgels showed superior inhibition of the expression than the simple mixture of MCC950 and the PPAD_3-7-1_ microgel, indicating the synergistic therapeutic effect against DED. As shown in Figure 5E, IL6, NLRP3, and IL-1β expressions at the protein level greatly increased after Scop treatment, while the MCC950@PPAD_3-7-1_ microgels markedly inhibited the expression of these inflammatory factors, which is well-consistent with PCR results.

## Conclusion

In summary, a synergistic strategy for topical treatment of DED was achieved through a polydopamine-based microgel loaded with the NLRP3 inhibitor, MCC950, targeting the ROS-NLRP3-IL-1β signaling axis. The PPAD microgels were successfully synthesized *via* copolymerization of three kinds of monomers. Due to the excellent adhesiveness of polydopamine, the drug bioavailability was significantly improved by prolonging the ocular surface retention time. The *in vitro* assay demonstrated that the polydopamine-based microgels exhibited good biocompatibility and ROS scavenging effect. Moreover, the potentially toxic effects of MCC950 at a high dosage can be avoided. TUNEL assay revealed that compared with free MCC950, PPAD microgels *in situ* loaded with MCC950 significantly reduced ocular surface epithelial cell damage and apoptosis. PCR and WB results indicated that both mRNA and protein expression levels of inflammation-related cytokines were also significantly inhibited. We believe that this work will provide insightful guidance in the synergistic treatment of inflammation-related diseases by targeting the ROS-NLRP3-IL-1β signaling axis.

## Data Availability

The original contributions presented in the study are included in the article/Supplementary Material, further inquiries can be directed to the corresponding authors.
