# Supplementary material for: Mussel-Inspired Microgel Encapsulated NLRP3 Inhibitor as a Synergistic Strategy Against Dry Eye
Source: Front Bioeng Biotechnol. 2022 Jun 1;10:913648. doi: 10.3389/fbioe.2022.913648 (PMC9198461; doi:10.3389/fbioe.2022.913648)
Supplement: Supplementary file 1 [file DataSheet1.docx]

***Supplementary Material***

**Supplementary Table 1**. Summary of the polymerizations of PPAD microgels

| Entry | Feed ratio *^a^* | Solids | *D*_h_ (nm)*^b^* | PDI *^b^* | Zeta potential *^b^* | Encapsulation efficiency *^c^* |
| --- | --- | --- | --- | --- | --- | --- |
| PPDA_5-5-1_ | 0.5/0.5/0.1/0.02 | 5% | 302 | 0.28 | +8.3 mV | 87.7% |
| PPDA_4-6-1_ | 0.4/0.6/0.1/0.02 | 5% | 334 | 0.24 | +10.7 mV | 88.7% |
| PPDA_3-7-1_ | 0.3/0.7/0.1/0.02 | 5% | 356 | 0.28 | + 15.9 mV | 88.3% |
| PPDA_2-8-1_ | 0.2/0.8/0.1/0.02 | 5% | 425 | 0.33 | + 22.7 mV | 89.0% |
| PPDA_1-9-1_ | 0.1/0.9/0.1/0.02 | 5% | 631 | 0.31 | + 46.7 mV | 84.6% |

*^a^* Molar ratio (PEGMA/APTAC/DPMA/LAP).

*^b^* Average value of the hydrodynamic diameter (*D*_h, app_), polydispersity index (PDI) and zeta potential of the PPAD microgels confirmed by DLS characterizations with the concentration of 0.1% (1 mg mL^-1^).

*^c^* Fluorescein sodium was adopted as a model molecule to confirm the encapsulation efficiency of PPDA microgels with feed ratio.


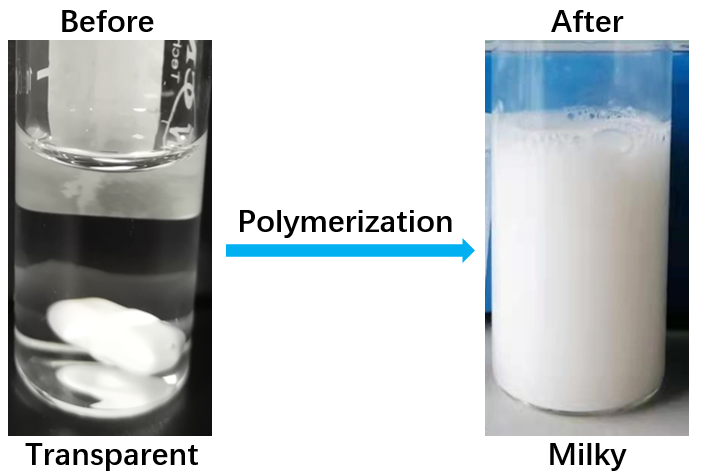


**Supplementary Figure 1**. The macroscopic images of the polymerization mixture before and after the injection of DPMA ethanol solution.

| 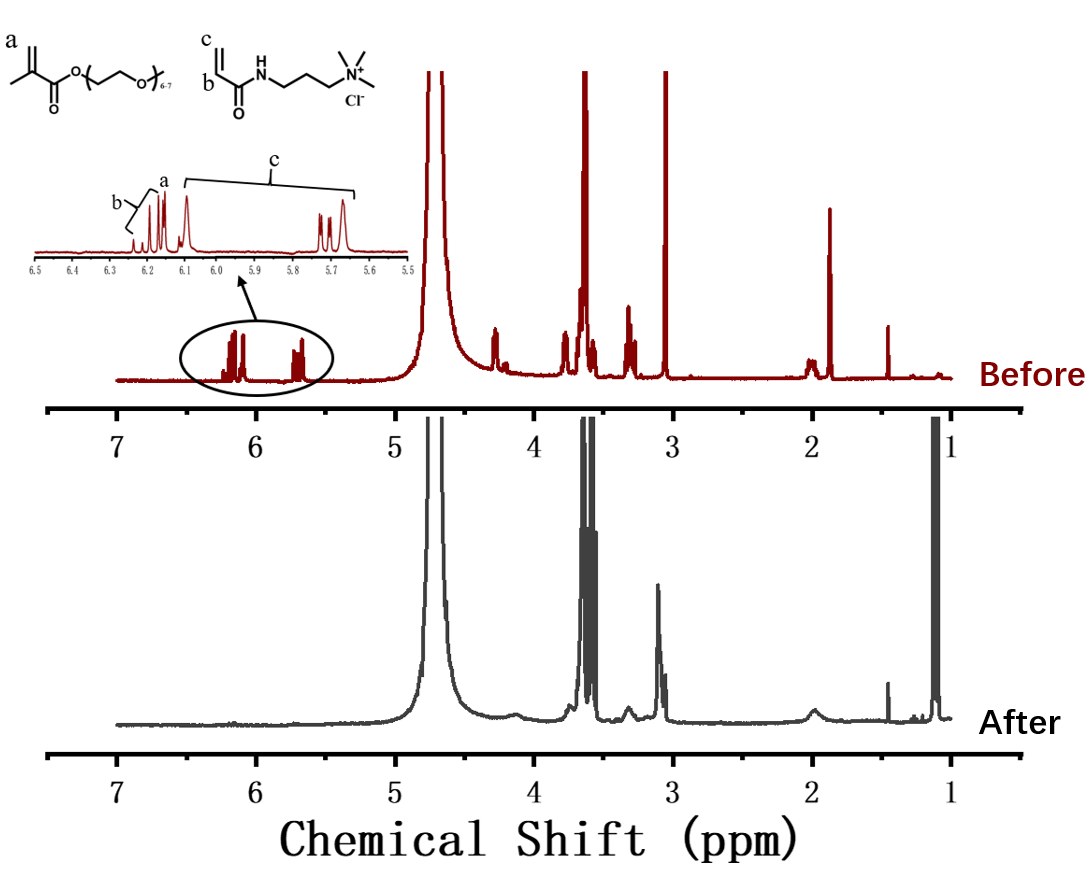  **Supplementary Figure 2**. ^1^H NMR spectroscopy of the mixed solution before and after polymerization. |
| --- |
| 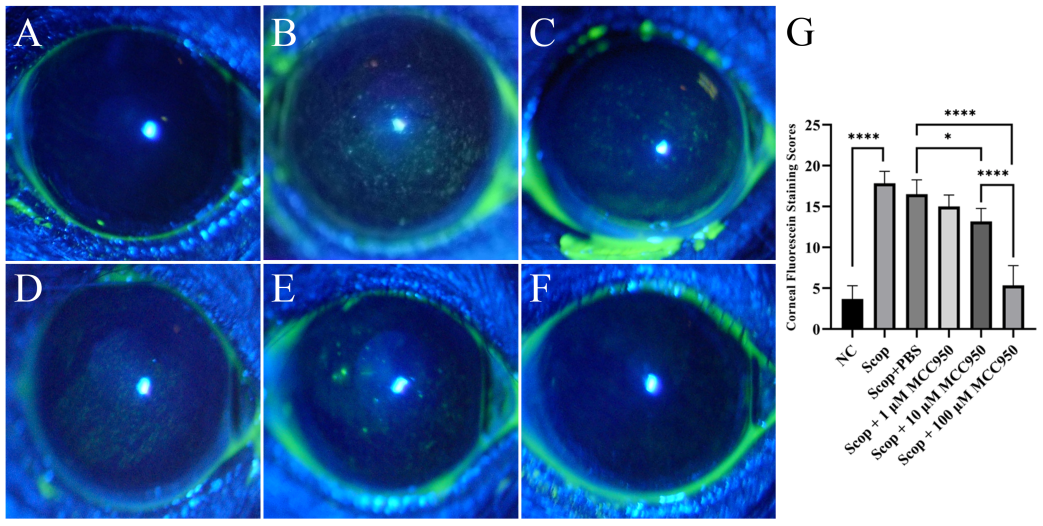 |
| **Supplementary Figure 3**. Corneal fluorescein sodium staining (CFS) scores of the experimental DE mouses treated with PBS, 1 μM MCC950, 10 μM MCC950 or 100 μM MCC950. (A) NC; (B) Scop; (C) Scop + PBS; (D) Scop + 1 μM MCC950; (E) Scop + 10 μM MCC950; (F) Scop + 100 μM MCC950. G) statistical result of CFS scores. Data are given as the mean ± SD (n = 6), **P*<0.05, *****P*<0.0001. |


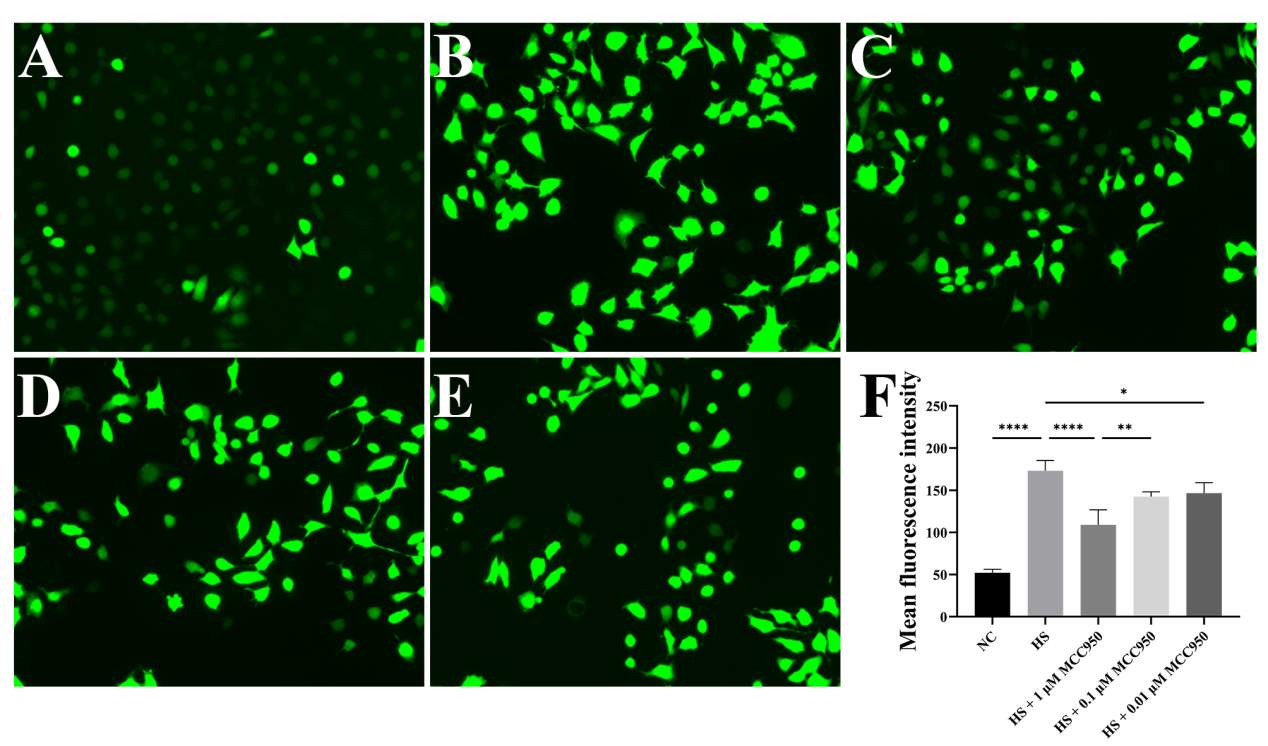


**Supplementary Figure 4.** In vivo ROS scavenging capacity of MCC950. (A): NC, (B) HS, (C) HS + 1 μM MCC950, (D) HS + 0.1 μM MCC950, (E) HS + 0.01 μM MCC950, (F) statistic result of the mean fluorescence intensity. Data are given as the mean ± SD (n = 5), **P*＜0.05, ***P*＜0.01, *****P*＜0.0001.


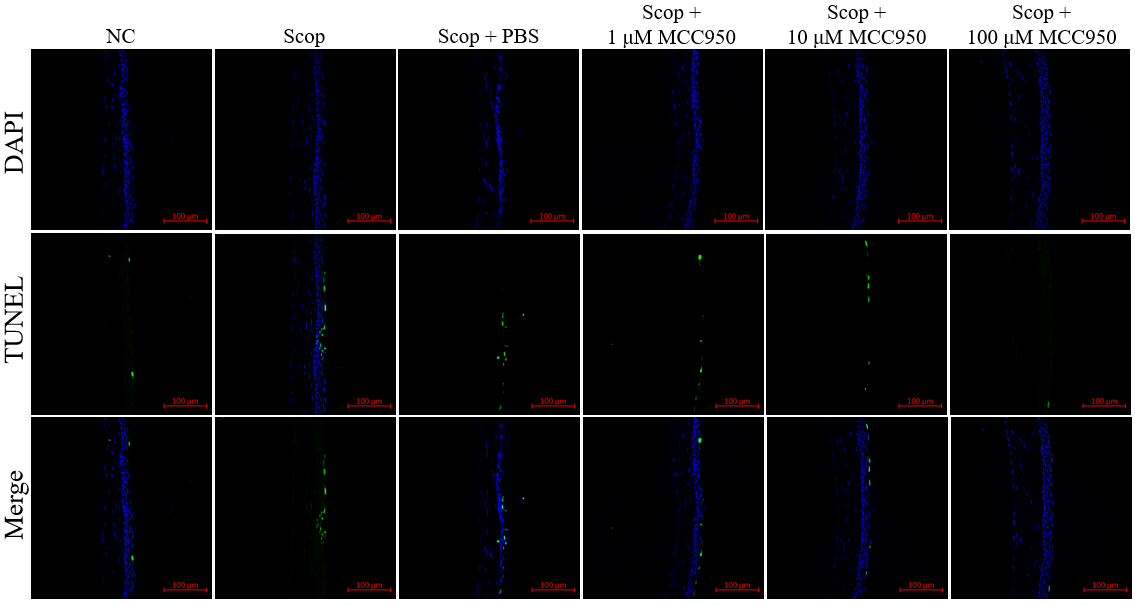


**Supplementary Figure 5**. The apoptosis of corneal epithelial cells in DE mice treated with PBS, 1 μM MCC950, 10 μM MCC950 or 100 μM MCC950 examined by TUNEL assay. Green fluorescence indicates TUNEL positive cells.


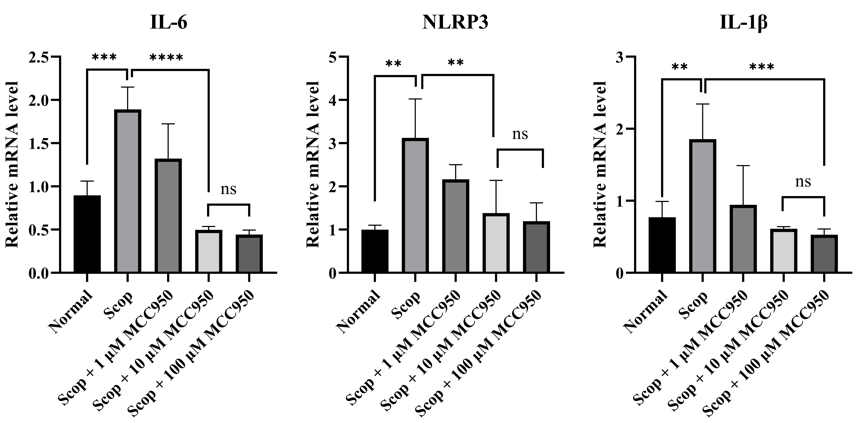


**Supplementary Figure 6**. The in vivo mRNA expression level of IL-1β, NLRP3 and IL-6 in DE mice treated with 1 μM MCC950, 10 μM MCC950 or 100 μM MCC950. Data are given as the mean ± SD (n = 4), ***P*<0.01, ****P*<0.001, *****P*<0.0001.

**Supplementary Table 2**. Primer sequences

| Primer (Mouse) | Segment | Sequence |
| --- | --- | --- |
| GAPDH (Glyceraldehyde 3-phosphate dehydrogenase) | Forward (5′-3′) | TGTCCGTCGTGGATCTGAC |
|  | Reverse (3′-5′) | CCTGCTTCACCACCTTCTTG |
| IL-1β (Interleukin-lβ) | Forward (5′-3′) | CACAGGAGCAACGACAAAATACCTGTG |
|  | Reverse (3′-5′) | TCTTCTTTGGGTATTGCTTGG |
| IL-6 (Interleukin-6) | Forward (5′-3′) | CTGATGCTGGTGACAACCAC |
|  | Reverse (3′-5′) | TCCACGATTTCCCAGAGAAC |
| NLRP3 (NOD-, LRR-and pyrin domain-containing protein 3) | Forward (5′-3′) | ATTACCCGCCCGAGAAAGG |
|  | Reverse (3′-5′) | TCGCAGCAAAGATCCACACAG |
